# Impact of Exclusion Netting Row Covers on ‘Honeycrisp’ Apple Trees Grown under Northeastern North American Conditions: Effects on Photosynthesis and Fruit Quality

**DOI:** 10.3390/insects10070214

**Published:** 2019-07-19

**Authors:** Gérald Chouinard, Jonathan Veilleux, Francine Pelletier, Mikael Larose, Vincent Philion, Valentin Joubert, Daniel Cormier

**Affiliations:** Laboratoire de Production Fruitière Intégrée, Institut de Recherche et de Développement en Agroenvironnement (IRDA), Saint-Bruno-de-Montarville, QC J3V 0G7, Canada

**Keywords:** organic agriculture, apple orchards, protected cultivation

## Abstract

Exclusion nets have been used successfully to protect fruit from insect pests of apples under various conditions, but the effect of each particular netting system on the plant itself has rarely been investigated. In this study, a complete exclusion system—in which the soil is also excluded—was used to grow ‘Honeycrisp’ apples for six years in southern Quebec, Canada. Abiotic conditions, as well as plant photosynthesis and fruit quality characteristics (colour, firmness, size, sugar content, number of seeds, ripeness and skin integrity) and yield were estimated yearly and compared in netted (either with or without a rainproof top) and unnetted row units. Although annual variations were high and results showed little or no difference between netted and unnetted rows for all measured variables, with the following exceptions; colour (increased red surface on fruits from unnetted rows some years), size (fruits from unnetted rows were smaller) and maturity (fruits from unnetted rows matured slightly faster). Fruits produced under nets had fewer microcracks at the surface than fruits produced without nets. Reduced cracking possibly helped decrease sooty blotch and flyspeck incidence and severity. Impacts for pest control and prospects for pesticide-free production are discussed.

## 1. Introduction

Nets are commonly used as an antihail device in tree fruit production [1,2]. Considering the various advantages they can provide to the grower (bird, insect, disease and mammal control and protection against hail, wind, frost and sunburn), they are also referred to as “multitask nets” [3]. More recently, nets are increasingly being considered for use as a nonaggressive pest control tool—being called exclusion nets in this context [4].

To date, the sole review [5] of the effects of agricultural nets on apple tree physiology and fruit quality focused on antihail and shading nets. Insect exclusion nets, which typically do not provide more than 10% shading, have been much less studied. Exclusion systems for pests of pome and stone fruits can be grouped into two categories, depending on whether the soil is excluded from the zone (complete exclusion) or not (incomplete exclusion) [1,2]. While incomplete exclusion can provide adequate pest control in many contexts, complete row-by-row exclusion currently appears more suited for the North American pest complex, which comprises three specific direct fruit pests, i.e., the tarnished plant bug, the plum curculio and the apple maggot [6]. Indeed, complete row-by-row exclusion has been the most studied of the two types of exclusion systems in northeastern north America [1,2]. Little research has focused on the quality of fruit grown under complete exclusion systems. In an Italian study [7], the authors measured fruit quality of apples and nectarines protected between petal fall and harvest by an incomplete row-by-row exclusion system designed to prevent damage from the brown marmorated stink bug *Halyomorpha halys* (Stål) (Heteroptera: Pentatomidae). They found no difference in colour, firmness or sweetness between fruit produced with or without exclusion nets, but for one apple cultivar (‘Baigent Brookfield’) they found higher amounts of anthocyanins in fruits from unnetted trees and higher numbers of polyphenols in the peel of fruits from netted trees. Results differed for the other cultivar (‘Galaval’), with differences observed only for polyphenols in the pulp (higher numbers in fruits from netted trees). It should be noted that these results are for an exclusion system made from antihail netting (mesh size: 2.4 × 4.8 mm), and that they concur with other studies [5,8], suggesting that changes in fruit quality under protective netting are often more influenced by the environmental conditions during a specific growing season than by the netting itself.

This paper presents the follow-up on a previous study which focused on the plant protection properties of a row-by-row exclusion netting system [1] for ‘Honeycrisp’ apples grown in northeastern North America. In that study, we hypothesised that this exclusion system, deployed over a high-density plot of high-valued apple trees, with no insecticide, acaricide or fungicide applications, would allow fruit and foliage growth with less damage than trees without netting. Results confirmed that hypothesis. Nets provided effective protection against the vast majority of key pests of apple, including the apple maggot *Rhagoletis pomonella* (Walsh), the tarnished plant bug *Lygus lineolaris* (Palisot de Beauvois) and the codling moth *Cydia pomonella* (L.). Protection was also significant for larger foliar pests, such as leafhoppers, while minimal or nonsignificant for lesser ones, such as aphids and mites (no flare-ups). Highly significant protection effects were also recorded for hail and mechanical damage from various causes. Unexpectedly, nets showed a light but significant protective effect from diseases such as apple scab *Venturia inaequalis* (Cooke) G. Wint., *Gymnosporangium* spp. rusts and sooty blotch and flyspeck (SBFS) complex.

Given the positive results concerning insects and mites, in the current study we report on the impact of this netting system on various photosynthesis and fruit quality parameters, evaluated over six years. We included an additional treatment in the experiment (rainproof exclusion, i.e., netting with a polyethylene cover) to verify if diseases could be excluded more effectively without adverse effects on quality, as proposed in a European study [9]. The impact of netting on the fruit cuticle was also investigated because of its role in gaseous exchanges, sunscreen and as a barrier against pathogens [10]. Fractures at different levels of the many layers of the cuticle are known to favour diseases [11], which can both alter fruit appearance and affect conservation. Cracks can be very superficial and do not necessarily reach the epidermal layer. Microcracking is in fact only visible through microscopy [12]. Since many environmental factors, including duration of moisture, contribute to microcrack formation [13], we hypothesised that netting could act as a barrier and reduce microcracking. This may partly explain why colonies of SBFS reported in the previous study [1] were less prevalent in units with netting or rainproof netting. It is possible that SBFS development could be favoured in the absence of netting, as microcracking could result in fruit leaking more sugars to the surface, which would benefit the fungi.

Our goal was to increase our understanding of the impact of pest exclusion netting on fruit quality (including cuticle integrity) by conducting an experiment with nets specifically designed for pest exclusion rather than hail protection (smaller mesh size, lighter colour), providing complete rather than incomplete exclusion. We hypothesised that our exclusion system would allow the trees to develop similarly and produce fruit of equal in quality to that of trees without netting.

## 2. Materials and Methods

Operations were conducted at the experimental orchard of the Research and Development Institute for the Agri-Environment (IRDA), located in Saint-Bruno-de-Montarville, QC, Canada (45°32’31” N, 73°20’30” W). The setup, which has been fully described [1], consisted of three blocks (l: 22 m; w: 14–28 m) of dwarf ‘Honeycrisp’ apple trees (h: 2–2.5 m) with six rows and two experimental units per row, each unit containing 8–12 trees (Supplementary Figure S1). All trees comprised within an experimental unit were subjected to sampling, except if stated otherwise in the “sampling” section. “Hobo” temperature and humidity sensors (Onset Co., Bourne, MA 02532, USA) were installed at a height of 1.5 m in the tree canopy to monitor hourly conditions in each experimental unit (in two of the three blocks, from 2014) [1,2].

In the first block, two treatments were compared from 2012 to 2017: exclusion (nets) and control (no nets); in 2013, the netted units also comprised a polyethylene rooftop (see below). Six pairs of the two treatments were distributed randomly within the 12 experimental units. In the two other blocks, three treatments were compared from 2015 to 2017: (1) exclusion nets, (2) rainproof exclusion nets (topped with polyethylene) and (3) no nets. Four replicates of each treatment were used, and treatments were allotted randomly within each block.

No insecticides, fungicides, acaricides or foliar fertilisers were sprayed in any of the experimental units after the nets were installed in the first year. Ground fertilisers and other agronomic practices (e.g., herbicide sprays, manual pruning and thinning) were applied uniformly in all treatments according to grower standards.

### 2.1. Exclusion System

The exclusion system (Supplementary Figure S2) was built following the methods fully described by [1], which makes use of the existing stakes and apple tree training and support system to support the netting. Lateral wooden studs and polyester wires are added to maintain the nets at a safe distance from the limbs, preventing damage to the trees and the nets. Clear high-density polyethylene nets (19 × 8 m; mesh size: 1.90 × 0.95 mm; 60 g/m^2^; 93% light transmission) (ProtekNet^®^, Dubois Agrinovation, Saint-Rémi, QC, Canada) are placed over each unit and closed ~30 cm above the ground with sturdy clips. This mesh size was selected to allow physical exclusion, based on the adult size of insect pests known to affect apple orchards in this part of the continent [14]. In the rainproof exclusion nets treatment, a 2.5-m-wide strip of polyethylene with vented flaps (thickness 6 mil; 82% light transmission) (Voen, Vöhringer GmbH & Co, Berg, Germany) was positioned centrally on top of netted units (Supplementary Figure S3).

The nets were installed between 22 April (bud break) and 13 May (pink stage) and, depending on the year, removed approximately one week before harvest and stored until reinstalled again the following spring. Netted units were opened each year during bloom, on either two (2014, 2015 or 2016), three (2017) or four (2012, 2013) occasions, i.e., on sunny days with fair weather, to allow between 20 and 40 h of effective pollination, mainly by honey bees from several hives deployed in the orchard. This amount of pollination hours was found to be sufficient to obtain good fruit set each year, i.e., at least one fruit set per 8 flowers, based on visual counts made prior and after bloom each year. On each of these “pollination days”, the bottom clips were detached in the morning, the nets rolled up and attached to the 189-cm-high wire on each side of the row, and then closed again at dusk to reduce the risk of entry by nocturnal pests. Nets were also detached for brief periods to collect data on several occasions during the summer. However, for these observations, nets were not lifted up but were left hanging down to the ground.

### 2.2. Photosynthetic Activity

The photosynthetic activity of trees in each experimental unit was obtained from two variables: the maximum photochemical efficiency of photosystem II, measured by the chlorophyll fluorescence (Fv/Fm ratio), which is a common method to compare plants stress between treatments, and the chlorophyll concentration as an overall indicator of plant health [15,16]. For these measurements, one tree was randomly selected from among representative ones in each experimental unit, and two shoots, one from each side, were chosen at mid-height of the tree. Sample clips (measurement area: 3 mm diameter circle) were placed on three leaves from these shoots (at bottom, mid-length and top of shoot) to create a sun barrier. After 30 min in the dark, a chlorophyll fluorometer (OS30p, Opti-Sciences, Hudson, NH, USA) was used to measure the chlorophyll fluorescence. Measurements for the chlorophyll content were made on the same leaves, although on an adjacent area not covered by the clips, using a chlorophyll content meter (CCM-300, Opti-Sciences, Hudson, NH, USA). In all cases, measurements were made at midday, on the adaxial side of the leaves and near the midrib. The ratio between the fluorescence at 735 nm and within the range of 700 to 710 nm (wavelengths used by the CCM-300) has been demonstrated to accurately estimate the chlorophyll content within leaves [15]. Both measurements were taken simultaneously on 1–3 occasions in 2012 (23 August), 2013 (12 August), 2015 (8 July, 7 August and 10 September), 2016 (23 June and 2 and 30 August) and 2017 (5 July and 24 August).

### 2.3. Fruit Quality

Fruit quality was evaluated yearly starting in 2012. At harvest time (~1–5 days prior to commercial picking), randomly chosen fruits were picked from five trees in each experimental unit. For each tree, apples were picked in equal numbers from both sides of the row. These apples were stored at 5 ± 1 °C for a maximum of five days until the assessments were completed. Of all fruits picked per experimental unit, 120 meeting the generally recognised market criteria were randomly chosen for evaluation. For each of these fruits, the colour and the number of seeds were evaluated. Between 2013 and 2014, apples were visually classified on a scale of 1 to 3, in which 1 = fewer than 30% red; 2 = between 30 and 50% red; and 3 = over 50% red, using a colour chart [17] based on the method originally developed by Nickerson [18]. In 2015, 2016 and 2017, fruit coloration was quantitatively measured through digital vision system image analysis (CV-X102, Keyence, Mississauga, ON, Canada) using a measurement algorithm developed to work with a digital colour camera (CV-035C, Keyence, Mississauga, ON, Canada). The algorithm converts images from RGB to CIE L*a*b* or CIE XYZ colour space format [19,20] and separates the total fruit area into two colour categories: cover colour (closer to red) and ground colour (closer to green) according to an adjustable green–red threshold. For this, each apple was placed on a turntable and a video camera was set to point at the fruit equator. A diffuse source of light was used to minimise reflection from the apple surface and to give a constant reading across the surface of the fruit. Camera and red colour range parameters were set as in Table 1. Cover colour values were measured on both sides (reddest, exposed side and greenest, shaded side) of the fruit and expressed as a percent of the total surface. The firmness, ripeness and sugar content of 15 fruits per experimental unit were measured. Firmness values were averaged from measurements on opposite sides of the fruit after peel removal, using a Magness-Taylor penetrometer [21] fitted with an 11 mm tip (FT 327, Facchini, Alfonsine, Italy). The ripeness was evaluated using the Cornell Starch Chart [22] by placing one apple half (cut along the equator) face down in an iodine solution and rating starch content on a scale of 1 to 8, in which 1 = 100% starch staining and 8 = 0% staining). For the sugar content, soluble solid concentration (°Brix) was measured using a hand-held digital refractometer [21] with temperature compensation (HI 96801, Hanna Instruments Inc., Woonsocket, Rhode Island, USA).

### 2.4. Fruit Cuticle Integrity

Cuticle integrity was measured on fruits picked on 23 September 2016 and 15 September 2017. In each experimental unit, 5–8 apples asymptomatic for SBFS were randomly picked from the top and bottom portions of trees from each unit. An area on the fruit shaded or exposed to light was circled with a marker pen on the equatorial and/or stem region, and the fruit was deposited in trays with the marked surface facing upward. A stainless steel washer (6.4 mm inner diameter, 17.8 mm outer diameter) was glued to one or two marked surfaces of each fruit and left to dry overnight. The washer cavity of each fruit was then filled with 100 µL of deionised water, and fruits were incubated for 2 h at room temperature (~21–22 °C). Water was then pipetted and analysed enzymatically (Megazyme) for the presence of sugar (d-fructose/d-glucose) and total sugar concentration was reported. Concentrations below 0.1 ppm were reported as zero. Then, for each unit, a subsample of one shaded fruit and one exposed fruit from the top of the trees was randomly selected. For each fruit, the peel circumscribed by the washers glued at both the equatorial and/or stem position was delicately cut away and filled with 100 µL of a solution of 0.1% acridine orange and 0.1% Silwet L-77 (Witco, Düsseldorf, Germany) in 50 mM citric acid (pH 6.5) [12]. After 2 min, fruit peels were blotted and the dyed fruit peel was removed from the washer with a scalpel. Fruit peel surfaces were observed under fluorescence microscopy (100×, excitation between 330 and 385 nm and emission at 420 nm) [13] and microcracking intensity was estimated on 10 fields of 621 × 468 µm per peel fragment using a nonparametric scale for both superficial thin wax cracks and deep wide cuticle cracking. The scale ranged from 0 (no cracking), 1 (a single crack smaller than epidermal cells), 2 (several small cracks or one crack longer than epidermal cells, 3 (fewer than eight large cracks) to 4 (abundance of cracking) [12].

In both 2016 and 2017, extra fruits with visible symptoms of SBFS were picked in each unit, rated for disease severity with a score (1 = few symptoms; 2 = moderate; 3 = severe), and analysed separately for sugar and microcracking as described above. Only wide cuticle cracks could be easily quantified in presence of disease. These fruits were used alongside the asymptomatic fruit to determine if sugar or cracking influenced SBFS severity. Finally, 180 fruits were randomly picked from each unit in 2017 and rated for SBFS as above to confirm previous year’s observations on the effect of netting on disease.

### 2.5. Fruit Load and Yield

Fruit size was evaluated between 2014 and 2017, and yield between 2015 and 2017. Yield was estimated as a function of fruit load, fruit size and mean fruit density, except in 2017 when it was directly measured by harvesting, counting and weighing all fruits. Fruit load was evaluated in late June, early July of each year following physiological fruit drop. For each experimental unit, six trees were chosen and, for each of them, the amount of fruits from 20 clusters (inflorescences) was noted as well as the number of fruit clusters in each tree. Towards harvest time in early September, the diameter of 20 apples from each of the selected six trees per experimental unit was measured at the equator of the fruits. The Vernier calliper used for the measurements was reset to zero once the observations on a single tree were completed and measurements for all experimental units were completed on the same day. Mean fruit density was estimated from yield measurements in 2017.

### 2.6. Analyses

Unless otherwise stated, all analyses were performed using R [23] with the lme4 package, and a linear mixed effect model (LMER) was used for the analysis of the relationship between treatments (unnetted, netted and netted + covered) and each measured variable (described above). As fixed effects, we entered the list of variables into the model one by one. To resolve nonindependencies, we choose to use blocks and years as random effects as well as by-block and by-year random slopes for the effect of treatments. Model confidence intervals were obtained using parametric bootstrapping (<1000). Assumptions for mixed models were taken in account using visual inspection of residuals by a Q-Q plot (which did not reveal any obvious deviations from homoscedasticity or normality). Likelihood ratio (LR) tests were used to compare the full model against the null model for which the chi-square statistics (χ^2^), degrees of freedom and associated *p*-value were reported. For the significative LR test, the partitioning of variance (repeatability) was calculated using conditional variances (pseudo R^2^) of the full model using the arm package [24]. Post hoc investigation and associated *p*-value of the model were done using the multcomp package [25] with a Tukey test and Holm adjustment.

For chlorophyll fluorescence ratio (Fv/Fm) we used a year-by-year beta regression model [26]. Heteroscedasticity was investigated with the studentised Breush and Pagan test [27]. Model selection was made using pseudo R^2^. LR tests were used to compare the final model against the null model for which χ^2^, degrees of freedom and associated *p*-value were reported. Improvement of the model fit was investigated using appropriate link function of the final model by log-likelihood differences. For red colour indices and number of set fruits, assumption for normality was not met therefore a Kruskal–Wallis test was used, followed by post hoc Dunn tests (with a Bonferroni correction) [28] to examine differences between treatments on a yearly basis.

For cuticle integrity, sugar was analysed as a presence/absence variable using binomial regression and the concentration was also analysed with normal regression. In both cases, the analysis was done as a generalised mixed effect model (GLMM) using unique combinations of rows and blocks as the random effects (seven levels). Similarly, cracking and SBFS scores were analysed with a cumulative link mixed model (CLMM) in the ordinal package of R [29]. Cracking and SBFS models were simplified as binomial when appropriate. Likelihood ratio test was used to compare model fit of fixed effects (treatment, year and position in trees) for which the chi-square statistics (χ^2^), the difference in the number of variables for the two models (degrees of freedom) and associated *p*-values were reported. For binomial regression and scores, the odds ratio (OR) of treatment effect relative to the control was calculated.

## 3. Results

### 3.1. Abiotic Conditions

**Figure 1:**
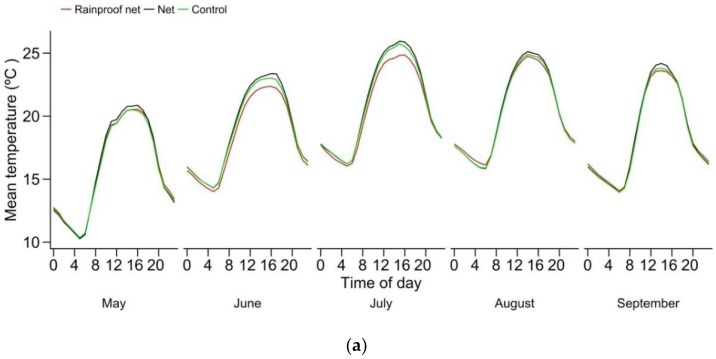

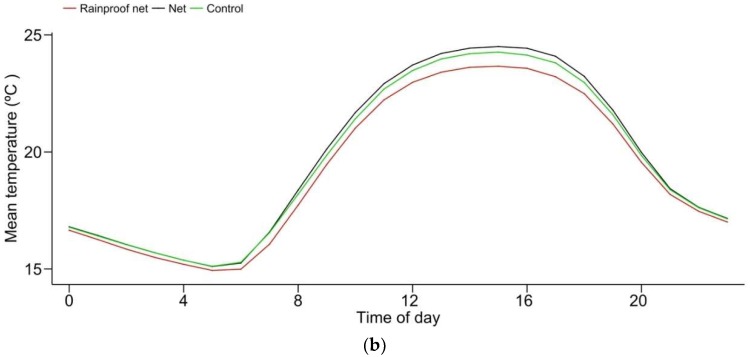
Mean hourly in-canopy air temperature (May–September 2012, 2013, 2015, 2016 and 2017): (**a**) averaged per month over years (**b**) seasonally averaged.

Slight, nonsignificant differences in temperature were observed among treatments (Figure 1). The presence of a rainproof polyethylene layer on top of the nets appeared to reduce the daily maximum temperature, but this difference was not significant. Average temperature overall between May and October was remarkably similar among treatments. The most important “heating effects” generated by our exclusion systems—as compared to unnetted units—were observed during daytime for units with nets only (<0,1 °C on average). The most important “cooling effects” were also recorded during daytime for units with rainproof exclusion nets (<0,25 °C on average).

**Figure 2:**
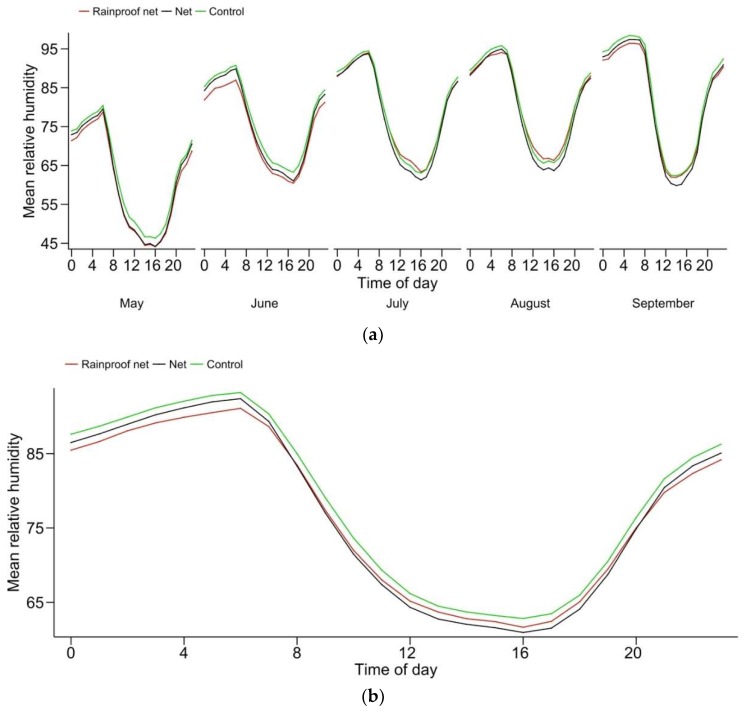
Mean hourly in-canopy relative humidity (May–September 2013, 2015, 2016 and 2017): (**a**) averaged per month over years and (**b**) seasonally averaged.

As was the case for temperatures, relative humidity conditions were similar among treatments (Figure 2). A significant effect of covered nettings was detected, however, humidity being slightly lower (~5%) throughout the day.

### 3.2. Photosynthetic Activity

Chlorophyll concentration did not differ among treatments, except in 2012 when concentrations were higher in controls compared to netted only treatments (Table 2). Differences in chlorophyll fluorescence (Fv/Fm ratio) were not significant except for two cases where slightly better results were obtained for trees under rainproof nets: vs. unnetted trees in 2013 and vs. trees under regular nets in 2016.

### 3.3. Fruit Quality

The effects of the two types of nets on fruit quality variables are presented in Table 3. Colour varied greatly among years. When colour was assessed qualitatively (2013 and 2014), fruits grown under nets appeared to develop less red colour than fruits in control units, but this was marginally significant in 2013 only. When colour was measured numerically (between 2015 and 2017), however, colour generally did not differ significantly among treatments (χ^2^(2) = 5.052, *p* < 0.08). Sugar content (as estimated by °Brix) varied significantly among years (χ^2^(2) = 38.008, *p* < 0.0001), but did not differ among treatments (χ^2^(2) = 0.245, *p* < 0.8849). The average number of seeds in each fruit was numerically higher in unnetted units than in other treatments in some years, and the model detected significant differences between the different treatments (χ^2^(2) = 7.231, *p* < 0.0269). Firmness also varied among years, but not among treatments (χ^2^(2) = 0.005, *p* < 0.9974). Fruit maturity evolved differently among treatments (χ^2^(2) = 6.714, *p* < 0.0348): the starch index of collected fruit was significantly higher (fruit less mature) in units with a rainproof net than in other treatments, in all years this treatment was evaluated. Maturity did not differ, however, among units with or without nets except in 2014, when fruits were again less mature in netted units.

### 3.4. Fruit Cuticle Integrity

**Figure 3:**
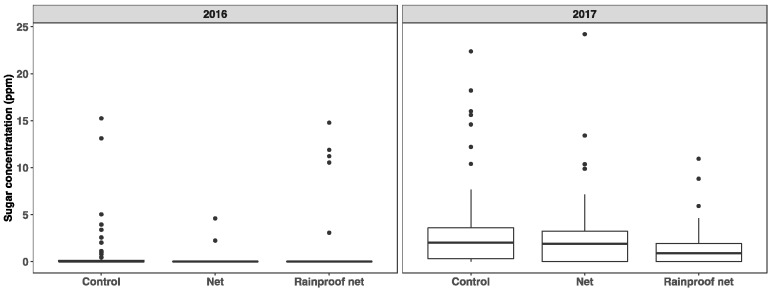
Sugar concentration at harvest on the surface of fruits from row units covered. With nets or rainproof nets compared to unnetted row units. The mid horizontal bar of the box represents the median, the two hinges correspond to the 25th and 75th percentiles. Whiskers extend 1.5× above and below the interquartile range. Data beyond the end of the whiskers were plotted individually.

Years (χ^2^ = 146(1), *p* < 0.0001) and treatments (χ^2^ = 8.5(2), *p* = 0.014) impacted the probability of finding sugar on the fruit surfaces (Figure 3). We found sugar on 27 and 77% of control fruits in 2016 and 2017, respectively (OR between the two years = 11), with average concentrations of 0.93 ppm and 3.4 ppm, respectively. The magnitude of the effect of netting treatments was different between years (interaction χ^2^ = 10.4(2), *p* = 0.006), but headed in the same direction. Fruits from units with rainproof netting were less likely (OR = 10 (2016), 2.8 (2017)) to have sugar on their surface compared to the control. Fruits from units with only netting were 10.9 times less likely to have surface sugar in 2016, but close to the control in 2017. The tree zone (top vs. bottom), fruit exposure (shaded or exposed side of fruit) or the location of sampling on the fruit (equatorial or near stem), did not modify the probability of finding sugar (χ^2^ < 1.2(1), *p* ≥ 0.28 for each). However, when modelled as the sugar concentration, treatment effects were similar but additionally we observed an overall increase of 0.64 ppm of sugars on fruits from the lower portion of the trees compared to the top (χ^2^ =3.6(1), *p* = 0.06). When considering only fruits positive for sugar, the increase was 1.7 ppm.

**Figure 4:**
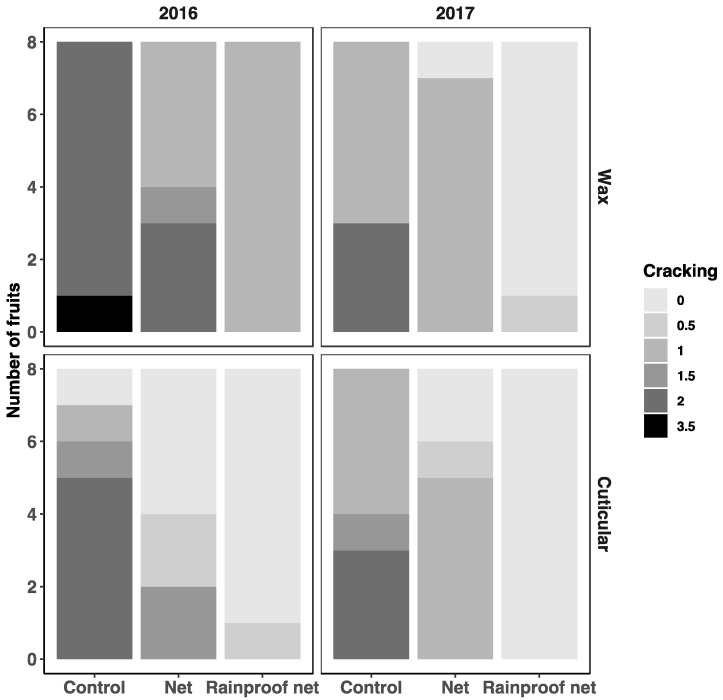
Cuticular and superficial wax cracking score at harvest on fruits from row units covered with nets or rainproof nets compared to unnetted row units. The graph represents the number of fruits for each cracking score, based on the median of 10 scores per fruit.

For surface (=wax) cracks (Figure 4), year (χ^2^ = 182(1), *p* < 0.0001), treatments (χ^2^ = 153(2), *p* < 0.0001) and their interaction (χ^2^ = 8.2(2), *p* = 0.02) impacted the odds of cracking. Higher crack intensity scores were less likely in control units of 2017 than in 2016 (OR = 4.5). In both 2016 and 2017, we observed less superficial cracks in the epicuticular wax on fruits from units with rainproof nets (OR = 8.9 and 22, respectively) compared to control units. Netted units without the rainproof cover had intermediate fruit cracking (OR = 3.1 in 2016 and 4.1 in 2017).

For deep cuticle cracks (Figure 4), a simple presence/absence binomial model gave the best fit to the data. Year (χ^2^ = 12(1), *p* = 0.0005), treatments (χ^2^ = 108(2), *p* < 0.001) and their interaction (χ^2^ = 32(2), *p* < 0.001) resulted in the most likely model. In both 2016 and 2017, we observed the fewest cuticle cracks on fruits from units with rainproof nets (OR = 3.7 and 34, respectively). Compared to control units, units with nets again had fruits intermediate for cracking (OR = 2.1 in 2016 and 11 in 2017), which was less than the control. Sugar at the surface and microcracking were not affected by orientation of the fruit in the tree or sampling location on the fruit.

Notwithstanding unit attribution, cuticular crack scores in each microscopic observation were positively correlated with wax cracks. (Kendall rank correlation tau = 0.41, *z* = 14.9, *p* < 0.001). Once adjusted for year, intensity of cuticular cracks on a given fruit was overall positively correlated with sugar presence. For each increase of one unit on the cracking scale for the maximal cuticle cracking observed, the odds ratio of finding sugar increased by 4.1 ((χ^2^ = 10 (1), *p* = 0.002). The relation between wax cracks and sugar was weaker (OR = 2, χ^2^ = 3(1), *p* = 0.08).

Similar to 2016 and previous years [1], in 2017, netting treatments again reduced SBFS incidence (χ^2^ = 63(2), *p* < 0.001) on fruit cuticle, and fruits from control units (30% incidence) were slightly more likely to be diseased than those from units with nets (OR = 2), but much more likely (OR = 210) than those from units with rainproof nets (incidence = 0.3%). With the combined SBFS data of the two years, we observed that disease severity increased with cuticle crack intensity. For each increase of one unit on the cracking scale for the mean cracking observed per fruit, the odds ratio of finding SBFS on that fruit was 5.9 ((χ^2^ = 19 (1), *p* < 0.001). Similarly, OR of SBFS for fruits positive for sugar was 2.7 ((χ^2^ = 4.1 (1), *p* = 0.04).

### 3.5. Fruit Load and Yield

Depending on the year, fruits from netted units and/or from units with rainproof nets were numerically larger (and heavier) than fruits from unnetted units (Table 4), which resulted in an overall significant difference between treatments for size (χ^2^(2) = 10.671, *p* = 0.0048) and weight (χ^2^(2) = 13.325, *p* < 0.001). This appeared to be the result of more fruit being pollinated in unnetted units, as fruit load, measured as the amount of fruit set per cluster, was frequently (three years out of six) significantly higher in unnetted trees than in trees under nets or rainproof nets. Yield varied among years but not among treatments (χ^2^(2) = 0,6297, *p* < 0.7299).

## 4. Discussion

### 4.1. Abiotic Conditions

Nets changed the temporal distribution of temperature fluctuations within the tree canopy, but did not change the overall average temperature, in accordance with other studies on protective netting for fruit trees [5] and on insect-proof netting for screenhouses [30]. Under the warmer growing conditions of Washington State (USA), antihail netting did not affect air temperature either [31]. However, since night temperatures were in general slightly warmer in rows with rainproof exclusion nets, heat units could start to accumulate earlier in the season, and night frost events were likely to be less frequent under that system, even though this “frost protection effect” was not very strong. It nevertheless was observed in 2012, when low temperatures in May damaged blossom petals (in unnetted rows only) and it can also explain our results in 2013 [1], when frost damage to harvested fruit was 93% higher in unnetted rows. Rainproof nets also generated a slight cooling effect at daily temperature peaks, which, when added to their nocturnal heating effect, can be characterised as a slight “buffer” effect.

Such a buffer effect was not apparent with nets only, under which measured air temperature was similar at night, and slightly higher at midday (mostly on warmest days) than temperatures in unnetted rows. The impact of these effects will be discussed more in the following sections.

Diurnal fluctuations in relative humidity followed very similar patterns in netted and unnetted rows. The observation published earlier that apple scab and other diseases develop slightly less under nets [1,32] can hardly be explained by altered humidity regimes, at least based on our data. Moreover, leaf wetness periods measured from the same units in 2015 [33] showed no difference in netted vs. unnetted units (~250 h). Another possibility for reduced expression of certain diseases on netted trees is discussed below (Section 4.4).

### 4.2. Photosynthetic Activity

Although photosynthetic activity, measured in our study by chlorophyll concentration and chlorophyll fluorescence, was similar four years out of five for both variables whether trees were netted or not, a reduction in photosynthetic activity is often assumed by growers who use exclusion systems, because the nets and polyethylene sheets intercept at least a small portion of the incoming light. In a recent review [5], authors identified many factors that can reduce photosynthesis under netted trees and suggested that a reduction in photosynthetically active radiation (PAR) could arise under nets of certain types (polyethylene vs. other material), mesh size and shape, thread thickness and colours. All these can change the quality of light passing through them by altering light diffusion (scattering), reflectance, transmittance and absorbance. Since the systems used in our study were characterised by 82 to 93% light transmission when new, and considering that these numbers should decrease by ~2% per year of field use [34], we had also assumed a reduction in PAR that would have been revealed by our chlorophyll measurements, but this was not the case. In one study [31], the authors reported that while netting did not affect air temperature, it greatly reduced fruit surface temperature during full sun and hot periods, because of the light scattering effects of the nets [35]. Scattering reduces direct exposure to radiation but improves light penetration into the plant canopy, thus increasing photosynthesis efficiency [36,37]. The light scattering effects of netting can be significant: for white antihail nets, a scattered/total light ratio of 23% was reported in the PAR range of 400 to 700 nm in a study [35]. The netting we used exhibited similar properties [38]: an average ratio of 26.2% ± 4.9 was obtained from seven measurements made a-posteriori using a ceptometer (SunScan SS1-UM-1.05, Delta-T Devices, Cambridge, UK). Light scattering possibly counterbalanced reduced light intensity in netted rows and resulted in an overall lack of effect of our netting system on tree photosynthesis. Enhanced efficiency of leaf-level photosynthetic light use, which has been reported for ‘Honeycrisp’ apple trees under photoselective shade nets [39], is not considered applicable to our conditions, because the reported effect is mostly apparent at high temperatures and under high solar radiation, which can be common in Washington state and in many apple growing areas but is not representative of northeastern North America.

### 4.3. Fruit Quality

The effects of netting on fruit quality can vary so much that the authors of a recent review [5] were unable to conclude that there was any pattern and suggested that changes in fruit quality under protective netting are more influenced by the environmental conditions in a specific growing season than the netting itself. Similarly, a consistent effect of the exclusion systems could not be detected for dependent variables considered in our study, except for the number of seeds per fruit, whose lower numbers in fruit from netted trees are highly related to reduced pollination rates in the exclusion systems (which are discussed below). The less consistent but still significant effects on fruit colour and maturity suggest a slowdown of these processes under nets—particularly the rainproof nets. Considering that high light intensity and cool temperatures are key factors for inducing red colour in apple [40], it is not unreasonable to hypothesise that slight reductions in light intensity combined with slight increases in maximum temperatures may be involved in those inconsistent effects. As for the starch breakdown in fruit (which is what is measured when estimating fruit maturity) our results support those of a Russian study [41] that reported a slowdown effect for ‘Pinova’ and ‘Fuji’ apples under many types of antihail nets; but studies on other cultivars [42,43] reported an increased starch breakdown (for ‘Gala’) or no effect (for ‘Cripps Pink’ and ‘Royal Gala’). Still, all these results apply to antihail nets, which differ from the nets in our study by their larger mesh size and usually darker colour.

### 4.4. Fruit Cuticle Integrity

Fruits from rows with rainproof exclusion nets were the least cracked, had the lowest sugar leakage, and showed the least SBFS for both years. Interestingly, while the netting treatment effects were similar for both years for both sugar and microcracks, the overall year effect was not: sugar leakage at the surface and SBFS increased in 2017 while cracks decreased. Since SBFS was positively correlated at the fruit scale with sugar presence and cracking, and since sugar was correlated with cuticular cracking in absence of apparent SBFS, it is likely that our original hypothesised mechanism for the reduction of SBFS observed in presence of rainproof nets is confirmed: in unprotected rows, increased cracking favours sugar leakage, which can favour SBFS. The sugar leakage may also be related to the delayed maturation of fruits under netting. The important yearly effect underlines other factors that may also influence cracking, sugar leakage, and disease appearance. Nonetheless, since microcracking can affect fruit storage and SBFS can be difficult to control, netting with a roof can be advantageous under northeastern North American growth conditions.

### 4.5. Fruit Load and Yield

Most previously published studies on this topic are again related to antihail nets or shade nets, which provide more shading (12–90%) rather than the ones used in our study (7–18%). The authors of a recent review [5] concluded that the effect of protective netting on fruit set in apple has been variable. Our data can shed little light on this since, although the netted trees we studied in general bore fewer, larger fruits, in all years the total yield was equivalent among treatments. It should be remembered, however, that pollination time was limited to the period when nets were left open. From this we conclude that the most important effects on fruit load and yield observed in our study appear more related to pollination (reduced number of seeds produced per fruit in the netted rows) than to the nets themselves and their reported variable effects on growth conditions.

## 5. Conclusions

Having been studied for six years and reported on in our previous [1] and current papers, the row-by-row complete insect exclusion system has, in our opinion, proven most of its expected benefits and limitations over conventional orchard pest control. Among its biggest benefits, it effectively prevents the entry of key fruit pests and reduces damage by species such as tarnished plant bug, codling moth, plum curculio and apple maggot, which could otherwise attack >90% the unprotected crop [6], to very low levels. Second, foliar pests (e.g., leafrollers, leafminers, leafhoppers and mites) are either similarly excluded, or in the case of smaller ones, allowed to develop in the pesticide-free environment provided, in which beneficial fauna is also allowed. In this context, with the exception of one species described below, no flare-ups have been reported for these pests over six years of experimentation. Third, as demonstrated in 2015 [1], the system provides full protection against hail, and also tempers the effects and damage from other abiotic factors such as strong winds and high temperatures. Fourth, it can also reduce disease (e.g., SBFS) incidence and severity, an effect that can be enhanced by the addition of rainproof lining over the trees, which has shown no special limitation (except for delayed maturity, which can be considered as either an advantage or a disadvantage for crop management). Reduced fruit cracking under nets and rainproof nets may also improve fruit storage, analysis of which is beyond the scope of this paper. Last, despite the fact that netting intercepts a portion of the incoming PAR, this generally did not significantly impact fruit quality and yield, based on the measurements made. Among current limitations, we can point out first that only ‘Honeycrisp’ apple trees have been thoroughly studied with this system, a cultivar that requires less protection from apple scab; a cultivar more susceptible to apple scab may also fare differently under netting and require specific sprays against this disease. Second, to be sustainable, there is a need to start with a clean orchard to prevent some species that have no requirement for metamorphosis outside the enclosures from developing high population levels under the protection of nets. Under current northeastern North American conditions, the only concerned species is the obliquebanded leafroller *Choristoneura rosaceana* (Harris) (Lepidoptera: Tortricidae). Other limitations include the reduced ability to spray through nets (for purposes such as fertilisation, fruit thinning and application of supplemental pesticides as needed), additional material and labour costs (mostly for net opening and closing) and reliance on fossil fuels for net production [44]. These limitations could be overcome by appropriate engineering and research, which should be pursued given the pesticide and climate change issues that need to be addressed in the near future.

These findings pave the way for using this exclusion system as an effective alternative, or addition, to integrated pest management (IPM) programs for orchards. Resistance to pesticides, reduced number of new pesticide registrations, increased pest problems caused by climate change and/or globalisation of commercial exchanges, societal requirements for pesticide-free products, etc. challenge current IPM programs. The availability of a pest control technique for orchards that overcomes these difficulties and that is not based on the usual paradigm—killing agents [2]—is, in our opinion, an essential tool to handle current and upcoming challenges in pest control worldwide. This is supported by the authors of recent reviews who stated that pest exclusion can be a very cost-effective and successful component of IPM for small-scale growers [4] to insure orchard sustainability in face of climate change issues and invasive exotic pests [45].

## Figures and Tables

**Table 1:** Camera and image parameters used for red colour measurement.

Parameters	Value or Interval
Shutter Speed	1/15
Sensitivity	4.0
Shift	10
Span	1.0
White Balance Coefficient ^1^	R 0.931; G 1.000; B 1.268
Hue	15–200
Saturation	0–255
Brightness	40–255

^1^ R: red; G: green; B: blue.

**Table 2:** Chlorophyll concentration and fluorescence ratio (mean ± SE, *n* = total number of observations) for apple leaves collected from row units without nets, with exclusion nets and with rainproof exclusion nets. Different letters indicate statistically significant differences between treatments for appropriate years (Tukey tests *p* < 0.005).

Year (*n*)	Control	Net	Rainproof Net
Chlorophyll concentration (mg/m^2^):			
2012 (36; 36)	682.42 ± 23.26 a	628.33 ± 23.71 b	
2013 (36; 36)	671.28 ± 21.23		711.22 ± 23.07
2015 (72; 72; 66)	795.85 ± 15.46	810.79 ± 15.69	822.83 ± 18.21
2016 (48; 48; 48)	667.58 ± 18.90	684.77 ± 20.37	719.54 ± 18.68
2017 (24; 24; 24)	752.17 ± 28.24	740.50 ± 30.56	791.54 ± 21.71
Fv/Fm ratio:			
2012 (36; 36)	0.78 ± 0.01	0.77 ± 0.02	
2013 (36; 36)	0.75 ± 0.06 a		0.76 ± 0.09 b
2015 (72; 72; 66)	0.78 ± 0.01	0.78 ± 0.00	0.79 ± 0.01
2016 (48; 48; 48)	0.78 ± 0.01 ab	0.76 ± 0.01 a	0.80 ± 0.00 b
2017 (24; 24; 24)	0.74 ± 0.02	0.71 ± 0.02	0.77 ± 0.01

**Table 3:** Fruit quality measurements (colour, soluble solids, number of seeds, firmness and maturity) (mean ± SE, *n* = total number of observations) for fruits harvested from row units without nets, with nets, and with rainproof nets. Different letters indicate statistically significant differences between treatments for appropriate years (Tukey tests except for nonparametrical red colour indices which required a Dunn test + Bonferroni correction, *p* <0.05).

Year (*n*)	Control	Net	Rainproof Net
Red colour index (2013–2014) or % red surface:			
2013 (120; 120)	2.26 ± 0.07 a		2.06 ± 0.07 b
2014 (80; 73)	1.75 ± 0.08	1.64 ± 0.09	
2015 (120; 120; 120)	47.74 ± 2.13	46.60 ± 2.60	43.11 ± 1.92
2016 (103; 120; 120)	37.89 ± 2.63	41.86 ± 2.18	32.05 ± 2.00
2017 (120; 120; 120)	47.95 ± 2.18	50.01 ± 2.16	32.92 ± 4.15
Soluble solids concentration (°Brix):			
2013 (60; 60)	12.79 ± 0.14		12.14 ± 0.10
2014 (79; 73)	12.01 ± 0.08	11.93 ± 0.08	
2015 (60; 60; 60)	11.84 ± 0.15	11.94 ± 0.16	11.91 ± 0.14
2016 (60; 60; 61)	12.36 ± 0.18	12.31 ± 0.12	12.31 ± 0.13
2017 (60; 60; 60)	11.72 ± 0.13	12.00 ± 0.14	12.00 ± 0.09
Number of seeds (per fruit):			
2013 (120; 120)	3.78 ± 0.21		3.53 ± 0.19
2014 (79; 73)	5.82 ± 0.23 a	4.89 ± 0.29 b	
2015 (120; 120; 120)	5.40 ± 0.21 a	4.35 ± 0.21 ab	3.83 ± 0.20 b
2016 (103; 120; 119)	6.94 ± 0.22	6.14 ± 0.21	6.07 ± 0.22
2017 (120; 120; 120)	7.18 ± 0.21 a	5.93 ± 0.23 b	4.91 ± 0.22 c
Firmness (kg):			
2013 (60;60)	6,24 ± 0.07		6.20 ± 0.08
2014 (79; 73)	8.01 ± 0.06	8.32 ± 0.07	
2015 (120; 120; 120)	6.37 ± 0.06	6.28 ± 0.06	6.31 ± 0.06
2016 (60; 60; 61)	7.05 ± 0.10	6.75 ± 0.06	6.57 ± 0.06
2017 (60; 60; 60)	7.07 ± 0.09	7.23 ± 0.08	7.36 ± 0.08
Maturity (% starch staining):			
2013 (60; 60)	20.57 ± 0.99 a		24.00 ± 0.99 b
2014 (79; 73)	48.43 ± 1.84 a	72.86 ± 3.25 b	
2015 (60; 60; 60)	13.09 ± 1.59 a	14.65 ± 1.80 a	28.10 ± 2.40 b
2016 (60; 60; 60)	25.29 ± 2.40 a	27.57 ± 1.69 a	33.86 ± 2.40 b
2017 (60; 60; 60)	12.43 ± 1.69 a	15.43 ± 1.84 a	41.15 ± 2.82 b

**Table 4:** Fruit load and yield measurements (number at fruit set, number removed during thinning operations, weight and size at harvest and weight per tree at harvest) (mean ± SE, *n* = total number of observations) for fruits harvested from row units without nets, with nets and with rainproof nets. Different letters indicate statistically significant differences between treatments for appropriate years (Tukey tests except for nonparametrical “set fruits” which required a Dunn test + Bonferroni correction, *p* < 0.05).

Year (*n*)	Control	Net	Rainproof Net
Set fruits (average number per cluster):			
2012 (720; 720)	1.35 ± 0.03 a	1.19 ± 0.03 b	
2013 (720; 720)	0.97 ± 0.04		0.98 ± 0.04
2014 (718; 412)	0.85 ± 0.03	0.77 ± 0.04	
2015 (480; 480; 480)	2.40 ± 0.05 a	2.22 ± 0.06 b	2.02 ± 0.05 b
2016 (480; 480; 480)	1.65 ± 0.04	1.64 ± 0.04	1.68 ± 0.04
2017 (456; 440; 426)	2.05 ± 0.04 a	2.10 ± 0.04 b	1.91 ± 0.04 c
Thinned fruits (number removed per tree):			
2015 (43; 46; 44)	60.42 ± 6.40	43.30 ± 4.70	26.07 ± 2.63
2016 (24; 24; 24)	44.71 ± 6.24	66.08 ± 6.57	53.58 ± 6.20
2017 (24; 24; 24)	85.00 ± 12.0	46.50 ± 9.63	36.33 ± 6.98
Fruit diameter at harvest (mm):			
2014 (80; 73)	76.22 ± 0.54 a	79.18 ± 0.67 b	
2015 (120; 119; 120)	86.33 ± 0.66 a	89.57 ± 0.60 b	91.62 ± 0.66 c
2016 (102; 120; 120)	78.39 ± 0.63 a	78.94 ± 0.52 a	84.59 ± 0.50 b
2017 (120; 120; 120)	81.38 ± 0.52 a	82.18 ± 0.52 a	85.94 ± 0.58 b
Fruit weight at harvest (g):			
2015 (120; 120; 120)	244.73 ± 5.40 a	269.33 ± 5.13 b	291.11 ± 5.76 c
2016 (103; 119; 120)	186.74 ± 4.25 a	189.74 ± 3.50 a	231.23 ± 4.13 b
2017 (120; 120; 120)	213.36 ± 4.04a	218.39 ± 4.32 a	249.99 ± 4.95 b
Estimated (2015–2016) or total yield (kg/tree):			
2015 (23; 24; 23)	17.09 ± 0.97	14.93 ± 0.98	13.98 ± 0.88
2016 (24; 24; 24)	12.45 ± 1.07	14.15 ± 0.67	16.10 ± 0.84
2017 (24; 24; 23)	11.87 ± 0.89	10.61 ± 0.84	9.63 ± 1.04

## Supplementary Materials

The following are available online at https://www.mdpi.com/2075-4450/10/7/214/s1, Figure S1: Experimental setup, Figure S2: Exclusion system, Figure S3: Net compared to rainproof net.
