# Supplementary figures and images for: Impact of Exclusion Netting Row Covers on ‘Honeycrisp’ Apple Trees Grown under Northeastern North American Conditions: Effects on Photosynthesis and Fruit Quality
Source: Insects. 2019 Jul 19;10(7):214. doi: 10.3390/insects10070214 (PMC6681268; doi:10.3390/insects10070214)

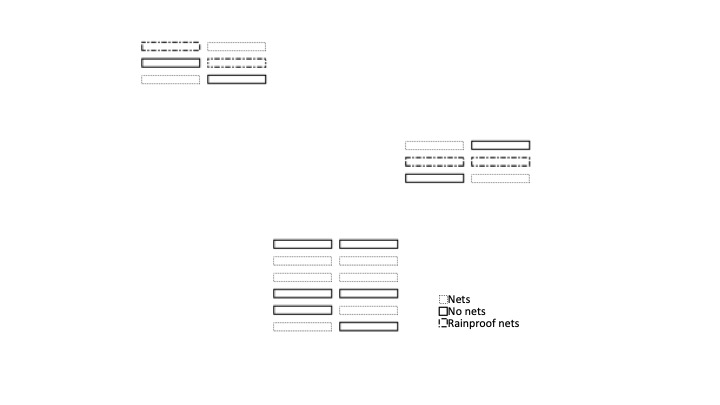

Supplement: Supplementary file 1 [file insects-10-00214-s001.zip › suppl/Figure S1-experimental setup.jpeg]

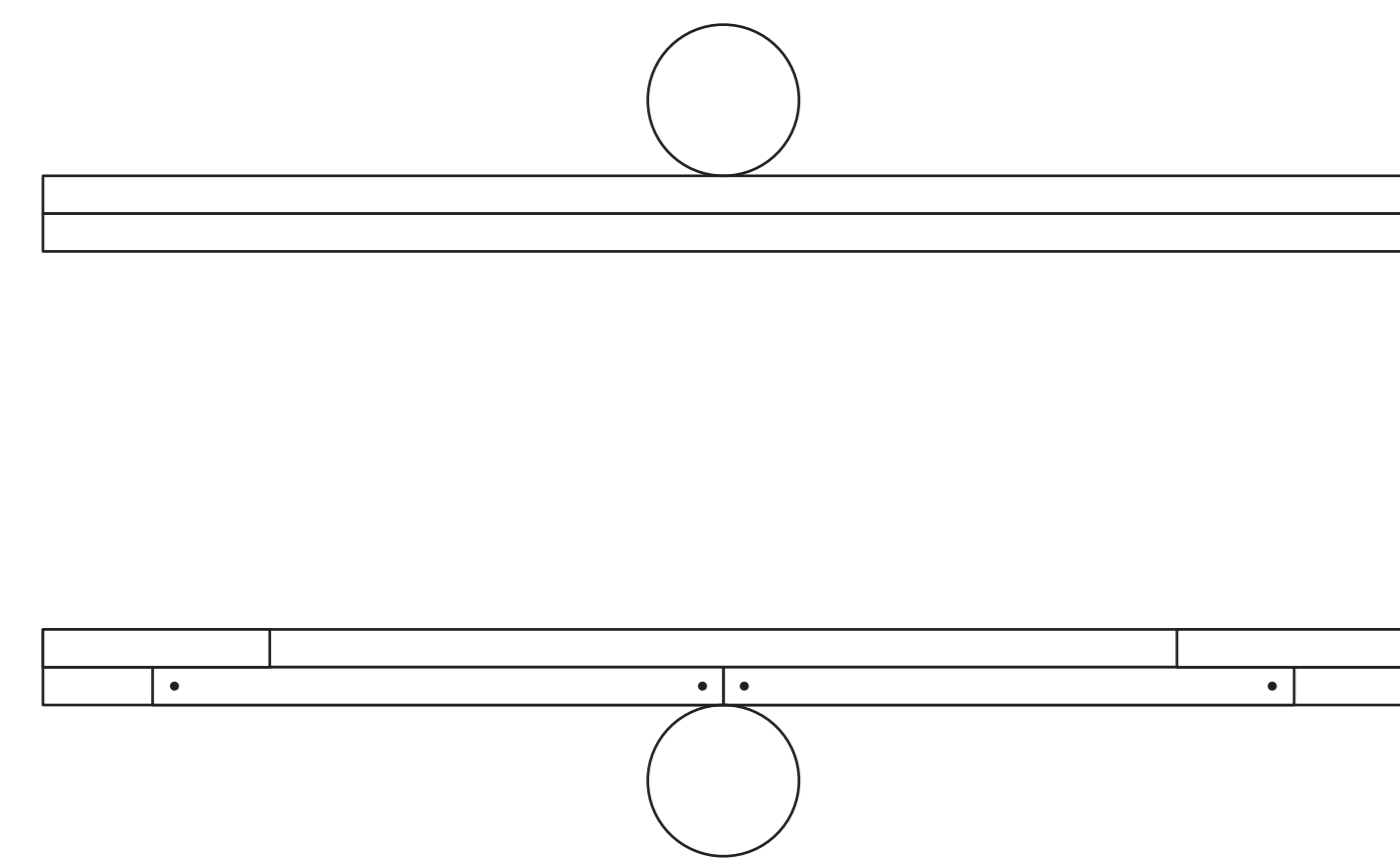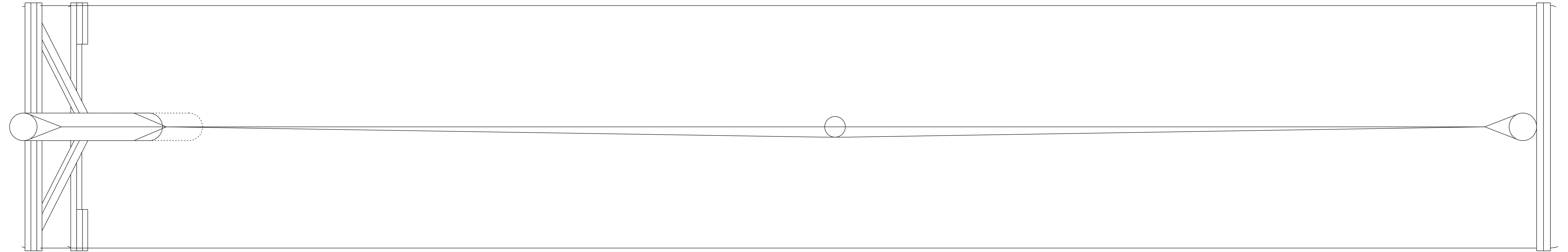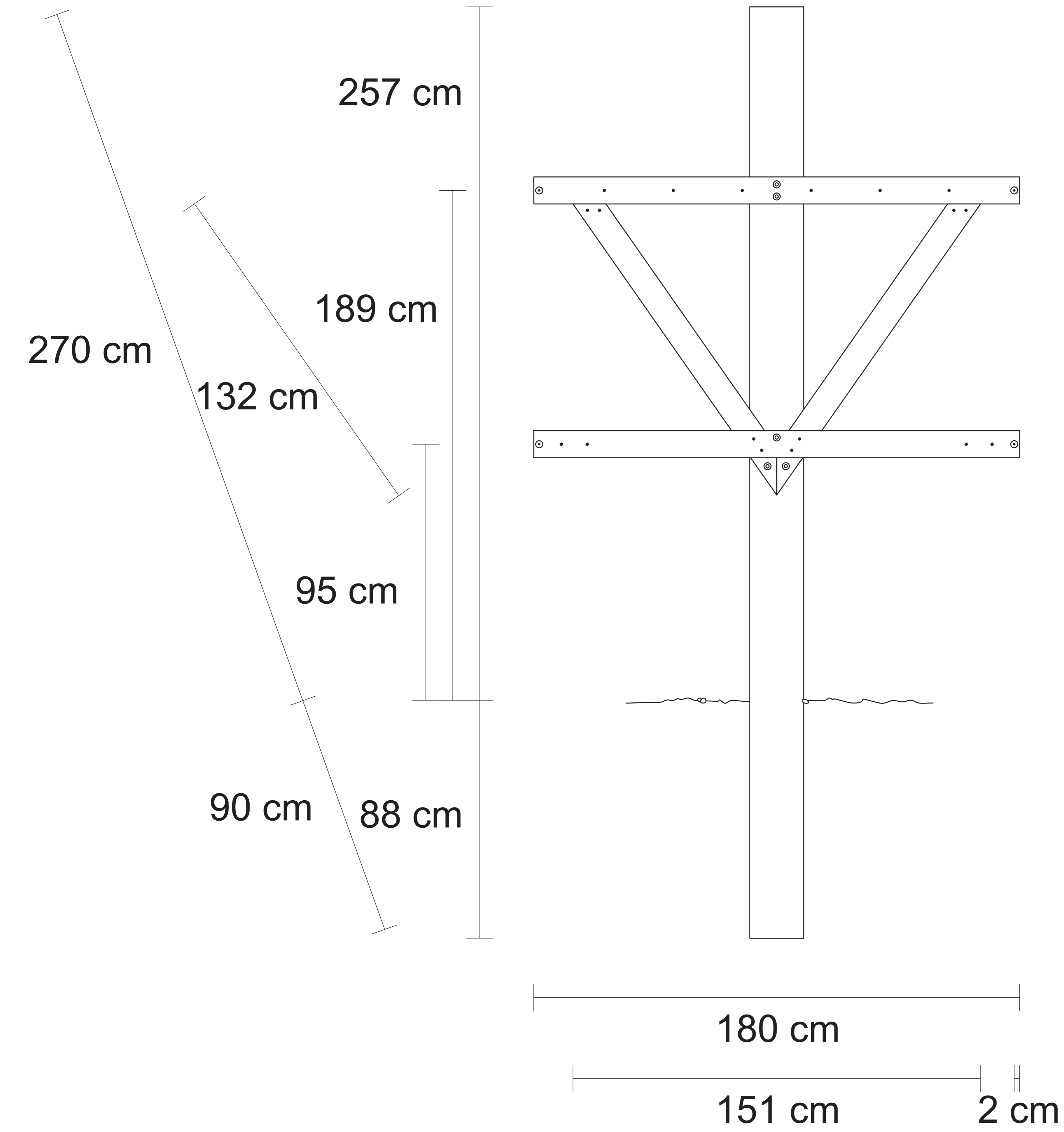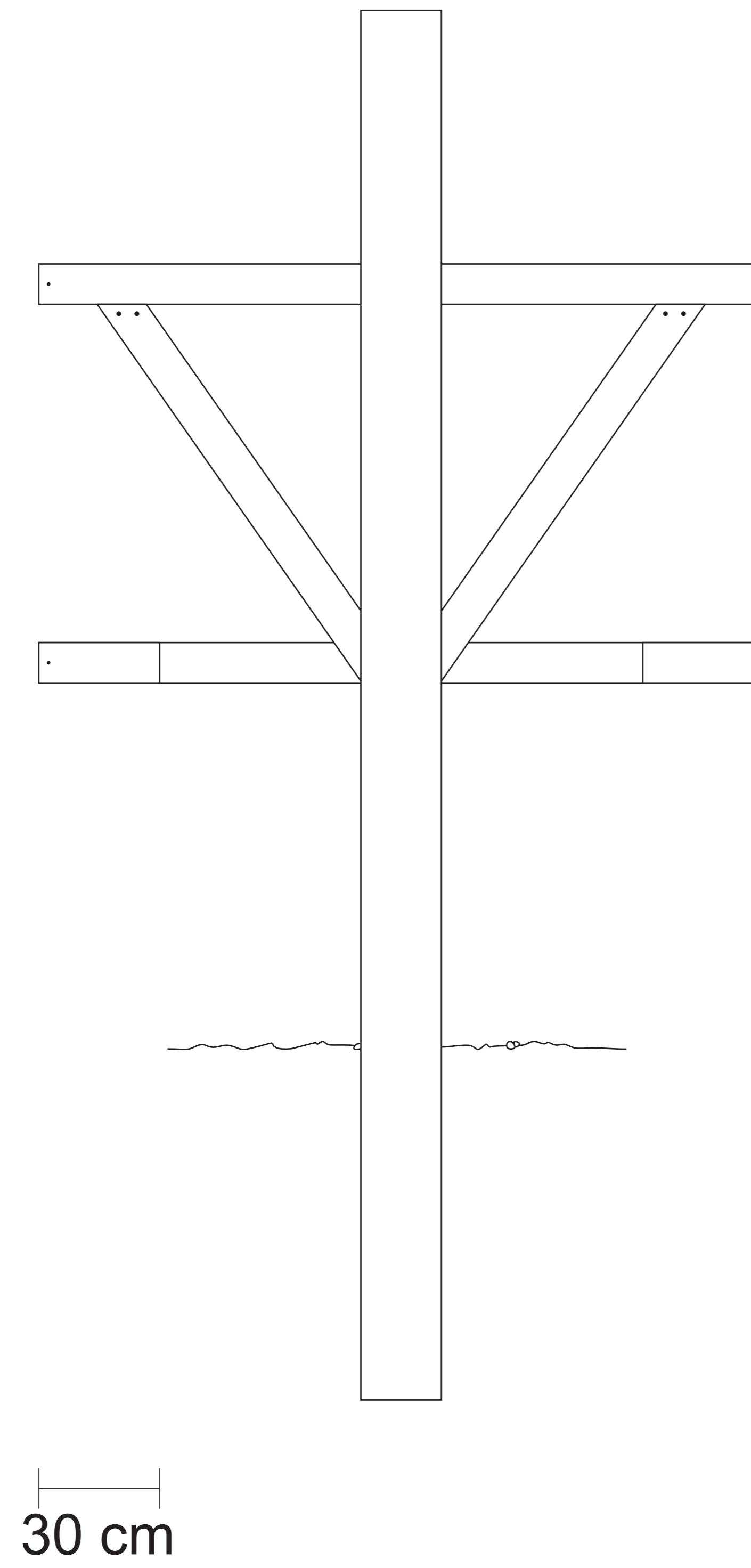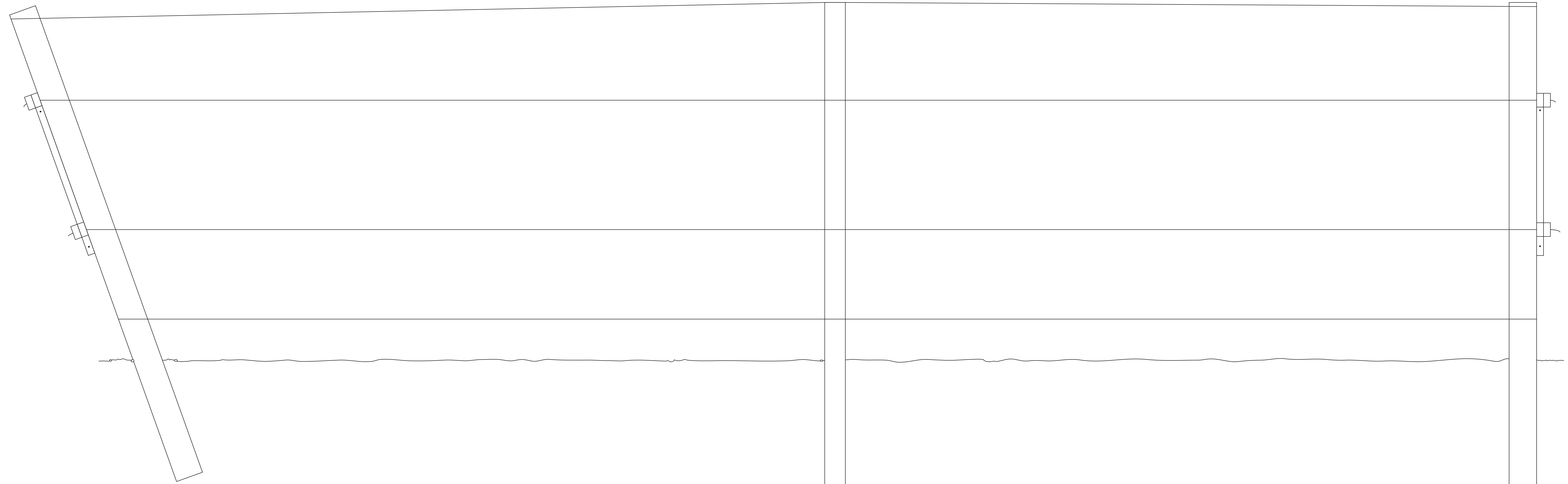

Supplement: Supplementary file 1 [file insects-10-00214-s001.zip › suppl/Figure S2-exclusion system.pdf]

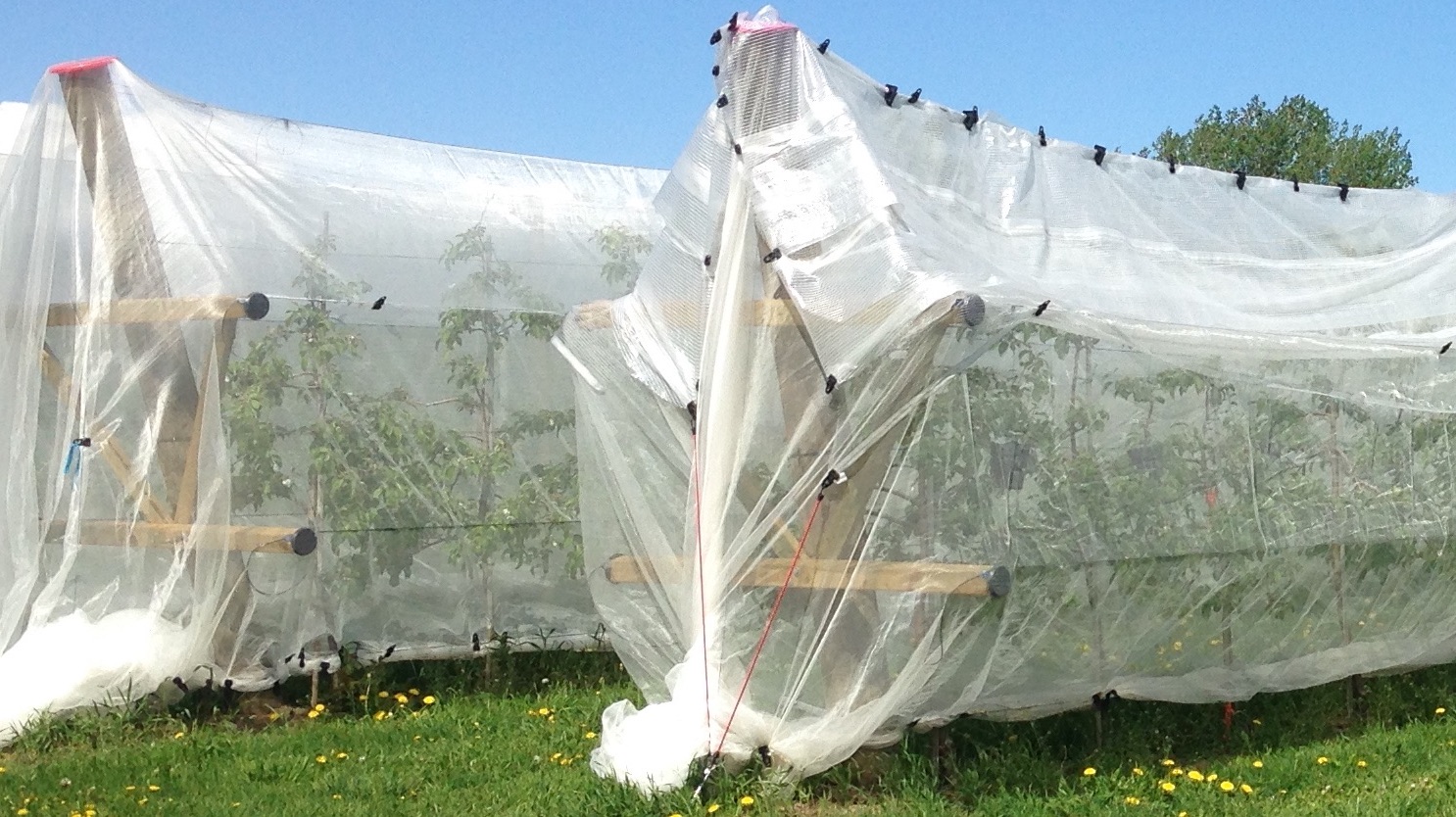

Supplement: Supplementary file 1 [file insects-10-00214-s001.zip › suppl/Figure S3-Net compared to rainproof net.jpg]
